# Re-endothelialisation after Synergy stent and Absorb bioresorbable vascular scaffold implantation in acute myocardial infarction: COVER-AMI study

**DOI:** 10.1186/s13063-019-3293-8

**Published:** 2019-04-11

**Authors:** Thibault Lhermusier, Paul Ohayon, Nicolas Boudou, Frederic Bouisset, Francisco Campelo-Parada, Jerome Roncalli, Meyer Elbaz, Didier Carrié

**Affiliations:** 1 Cardiovascular and Metabolic Pole, CHU Toulouse Rangueil, University Paul Sabatier, Toulouse, France; 2 Department of Cardiology, Rangueil Hospital, 1 avenue du Pr. J. Poulhes, 31059 Toulouse Cedex, France

## Abstract

**Background/aims:**

Drug eluting stent (DES) decrease the risk of restenosis by reducing the neointimal response. However, DES may impair strut coverage, and this has been associated with late stent/scaffold thrombosis. Bioresorbable vascular scaffold (BVS) may overcome the risk of stent/scaffold thrombosis when completely resorbed. The purpose of this randomised trial was to compare the arterial healing response in the short term, as a surrogate for safety and efficacy, between the metallic everolimus-eluting stent (Synergy; Boston Scientific, Marlborough, MA, USA) and the everolimus BVS (Absorb; Abbott Vascular, Santa Clara, CA, USA) in the particular setting of acute myocardial infarction (AMI). This pilot study sought to compare the neointimal response of metallic everolimus DES (Synergy) with polymeric everolimus BVS (Absorb) by optical coherence tomography (OCT) 3 months after an AMI.

**Methods:**

COVER-AMI was a single-centre, single-blind, non-inferiority, randomised controlled trial. Patients with ST segment elevation myocardial infarction (STEMI) who underwent primary percutaneous coronary intervention were randomly allocated (1:1) to treatment with the Synergy DES or Absorb BVS. The primary endpoint was the 3-month neointimal response assessed as the percentage of uncovered struts, neointimal thickness, in-stent/scaffold area obstruction, and pattern of neointima. The main secondary endpoint included the device-oriented composite endpoint according to the Academic Research Consortium definition.

**Results:**

Twenty patients without clinical and/or angiographic complications (Synergy (*n* = 10) or BVS (*n* = 10); mean age 59.0 years; 20% female) were enrolled in our centre. The stent diameter was higher in the Synergy group (3.7 ± 0.4 mm vs 3.4 ± 0.4 mm in the BVS group, *p* = 0.01). At 3 months, no significant differences in angiographic lumen loss were observed between the everolimus DES and everolimus BVS (0.04 mm (IQR 0.00–0.07) vs 0.11 mm (IQR 0.04–0.31), *p* = 0.165). OCT analysis of 420 cross-sections showed that the total neointimal area and in-stent obstruction were lower in the Synergy group, while OCT analysis at the strut level (*n* = 3942 struts) showed that the rate of uncovered struts was lower in the BVS group.

**Conclusions:**

Stenting of culprit lesions in the setting of STEMI resulted in a nearly complete arterial healing for both the Synergy and the BVS devices. Lower neointimal thickness and in-stent obstruction but a higher rate of uncovered struts were observed in the Synergy group. These findings provide the basis for further exploration in clinically oriented outcome trials.

**Electronic supplementary material:**

The online version of this article (10.1186/s13063-019-3293-8) contains supplementary material, which is available to authorized users.

## Background

Incomplete re-endothelialisation following stent implantation is strongly associated with stent thrombosis. Optical coherence tomography imaging (OCT) revealed an increased frequency of uncovered and/or malapposed stent struts, residual thrombus, and late pathological remodelling in lesions of ST segment elevation myocardial infarction (STEMI) compared with stable coronary artery disease patients at mid-term and long-term follow-up [1–3]. To date, only one OFDI study reported the arterial response in a head-to-head comparison between Absorb and everolimus-eluting stent (EES), in stable coronary artery disease [4]. One year after implantation, the neointimal thickness and percentage in-device area obstruction were comparable between groups. On the other hand, the vascular response after Absorb BVS implantation seems similar to that observed with Xience EES at 6 months in this particular setting of STEMI [5] but data concerning early re-endothelialisation remain scarce. The Synergy EES, which is now widely used, is made with biodegradable PLGA polymer, a platinum chromium scaffold, and thinner struts than the Xience and showed a particularly rapid endothelialisation in non-randomised preclinical studies [6]. The purpose of this randomised, controlled, prospective pilot study was to compare the neointimal response of the Synergy EES and the Absorb BVS by OCT at 3 months after acute myocardial infarction (AMI).

## Methods

### Patients’ enrolment and study design

The study design and protocol have been executed according to the SPIRIT 2013 Statement (see Additional file 1 and Additional file 2: Figure S1). The study included patients presenting with STEMI with the following ECG criteria: at least 1 mm in two or more standard leads or at least 2 mm in two or more contiguous precordial leads, within the first 12 h after symptom onset, requiring emergent percutaneous coronary intervention (PCI) with a vessel size ranging between 2.25 and 3.8 mm and following adequate lesion preparation. The main exclusion criteria included cardiogenic shock, severe tortuosity, or calcification and inadequate vessel size (< 2.25 or > 3.80 mm). All patients were randomised 1:1 to one of two treatment arms (Synergy vs Absorb stent) using a sealed envelope technique. This study is an exploratory pilot study, with no hypothesis regarding the expected difference in strut coverage between the two groups. Hence, a number of 10 patients in each group has been arbitrarily set.

Randomisation was performed after establishment of at least TIMI 2 flow using sealed envelopes. Written informed consent was required and obtained from all patients prior to randomisation. Randomisation was performed by dedicated web-based software. Patients were blinded to the treatment. Our study received approval from our Medical Ethics Committee. The study was conducted in compliance with the protocol, the Declaration of Helsinki, and applicable local requirements.

### Study endpoints

The primary endpoint was the 3-month neointimal response assessed as the percentage of uncovered and/or malapposed struts, neointimal thickness, in-stent/scaffold area obstruction, and pattern of neointima [7–9]. For the coronary optical frequency domain imaging (OFDI) endpoint analysis, the stent area and derived measures were based on the abluminal stent contour [10, 11]. The main secondary clinical endpoints included device-oriented composite endpoint (DOCE; composite of cardiac death, target vessel myocardial infarction (MI), and clinically driven target lesion revascularisation (TLR)) at 3 and 12 months; the individual components of DOCE; device and procedural success, all-cause death; any myocardial infarction; non-clinically driven TLR; clinically indicated and non-clinically driven target vessel revascularisation; and stent thrombosis, as defined by the Academic Research Consortium [12]. Reinfarction is defined according to the Third Universal Definition of MI as evidence of myocardial necrosis in a clinical setting consistent with acute MI [13]. Device success was defined as the implantation of the assigned study device with post-procedure residual stenosis < 30%. Procedure success was defined as device success without the occurrence of any component of the DOCE. Clinical follow-up was scheduled at 3 and 12 months. Angiographic follow-up was scheduled at 3 months.

### Percutaneous coronary intervention procedure

Primary PCI and stent implantation were carried out in accordance with current standards [14]. The Absorb stent was available in diameters of 2.5, 3.0, and 3.5 mm and in lengths of 8, 12, 18, and 28 mm. It was recommended to use similar sizes for the EES (Synergy; Boston Scientific). It was recommended that patients received a loading dose of aspirin and a P2Y12 inhibitor pre-procedure, followed by dual antiplatelet therapy for at least 12 months.

### Angiographic and optical frequency domain imaging analysis

Angiographic endpoints at 3 months included percent diameter stenosis, minimal lumen diameter (MLD), and late lumen loss. All angiographic endpoints were assessed for the in-segment, in-device, proximal, and distal region. Optical frequency domain imaging endpoints were assessed at 3 months and included all individual components of the healing, the mean and minimal stent diameter, area and volume, the frequency of incomplete strut apposition including area and volume, the percentage of uncovered struts, the mean neointima thickness together with the neointimal hyperplasia area on top of the strut and inter-strut and volume, the mean flow area and volume, and the intraluminal defect area and volume.

Optical frequency domain imaging assessment of the stented coronary segment was performed using the Saint Jude console and the FastView catheter. Angiography and OCT recordings were sent to an independent Core Laboratory (Zwolle, the Netherlands) for off-line analysis.

### Statistical analysis

Statistical analyses were performed using SPSS software, version 20.0 (SPSS Inc., Chicago, IL, USA), and Stata Software (Stata Statistical Software Release 10, College Station, TX, USA). Discrete variables are presented as counts and percentages, and continuous variables as means ± standard deviation (SD) when normally distributed and as median (interquartile range (IQR)) when non-normally distributed. Normally distributed data were compared using one-way analysis of variance or *t* tests, and non-normally distributed data were compared using the Mann–Whitney test. Categorical data were compared using Fisher’s exact test or the chi-square test. A two-tailed *p* value of 0.05 was considered statistically significant.

### Role of the funding source

The trial was designed by the principal investigator. The trial was supported by unrestricted grants from Boston Scientific Corporation. The investigator funded an independent data management and analysis centre (Diagram, Zwolle, the Netherlands) for database management and all statistical analyses.

## Results

### Baseline clinical characteristics

A total of 22 lesions (12 treated with everolimus DES and 10 treated with everolimus BVS) in 22 patients was selected for the present study. Two patients in the Synergy group were excluded before 3-month OCT assessment for stent thrombosis and renal failure. All non-clinical and clinical outcomes data were collected from the 20 remaining patients. Baseline clinical characteristics were comparable among both groups (Table 1). Procedural characteristics are presented in Table 2. Thrombectomy was used in two patients (one in each group) and pre-dilatation was attempted in 65% of the population (9 out of 10 in the Absorb group vs 4 out of 10 in the Synergy group) without significant difference between the groups. Post-dilatation was more frequently performed in the Absorb arm (80 vs. 10%, *p* < 0.005). On average, a total of 1.05 stents was implanted at the culprit lesion with a mean total length of 22.6 mm (± 5.3). The mean nominal diameter was larger in the Synergy arm (3.7 vs 3.4 mm, *p* = 0.01).

**Table 1:** Baseline clinical parameters

	Absorb (*n* = 10)	Synergy (*n* = 10)	*p*
	*N* (%)	*N* (%)	
Age (years)	56.5 ± 13.6^a^	61.4 ± 9.0	0.350
Male	9 (90.0)	7 (70.0)	0.582
Treatment for hypertension	3 (30.0)	3 (30.0)	1.000
Treatment for hypercholesterolemia	2 (20.0)	0 (0.0)	0.474
Diabetes mellitus	1 (10.0)	0 (0.0)	1.000
Current smoking	5 (50.0)	4 (40.0)	1.000
MDRD clearance < 60 ml/min/1.73 m^2^	1 (10.0)	1 (10.0)	1.000
Fasting plasma glucose ≥ 7 mmol/l	3 (30.0)	8 (80.0)	0.070
LVEF (%)	52.5 ± 7.9	52.9 ± 10.3	0.923
LAD significant lesion (> 50%)	9 (90.0)	7 (70.0)	0.582
Cx significant lesion (> 50%)	5 (50.0)	1 (10.0)	0.141
RCA significant lesion (> 50%)	5 (50.0)	8 (80.0)	0.350
> 1-vessel disease	8 (80.0)	6 (60.0)	0.628

*Cx* circumflex artery, *LAD* left anterior descending, *LVEF* left ventricular ejection fraction, *MDRD* Modification of Diet in Renal Disease, *RCA* right coronary artery

^a^Mean ± standard deviation. *t* test used for comparisons

**Table 2:** Procedural characteristics

Index PCI	Absorb (*n* = 10)	Synergy (*n* = 10)	*p*
	*N* (%)	*N* (%)	
Pre-dilatation	9 (90.0)	4 (40.0)	0.057
Post-dilatation	8 (80.0)	1 (10.0)	0.005
Vessel diameter (mm)	3.4 ± 0.5^a^	3.7 ± 0.4	0.118
Vessel < 3.5 mm	3 (30.0)	1 (10.0)	0.582
Implanted stents			1.000
One	9 (90.0)	10 (100)	
Two	1 (10.0)	0 (0)	
Stent(s) diameter	3.3 ± 0.4	3.7 ± 0.4	0.010
Stent(s) < 3.5 mm	4 (40.0)	1 (10.0)	0.303
Stent(s) length	23.7 ± 5.2	21.6 ± 5.4	0.388

*PCI* percutaneous coronary intervention

^a^Mean ± standard deviation. *t* test used for comparisons

### Procedural and quantitative angiographic characteristics

Angiographic characteristics are presented in Table 3. The interpolated reference vessel diameter and diameter stenosis were higher in the Synergy arm with borderline significance. Post implantation, MLD was higher in the Synergy group (2.99 vs 2.41 mm, *p* = 0.02). At 3-month follow-up, the lumen loss tended to be lower for the Synergy device but the difference was not significant (0.03 mm (IQR 0.00–0.07 mm) vs 0.11 mm (IQR 0.04–0.31 mm), *p* = 0.165).

**Table 3:** Quantitative coronary arteriography (QCA) results post implantation and at 3-month follow-up

	Overall (*n* = 20)	Absorb (*n* = 10)	Synergy (*n* = 10)	*p*
	*N* (%)	*N* (%)	*N* (%)	
Vessel				0.141
RCA	12 (60%)	4 (40%)	8 (80%)	
LAD	6 (30%)	5 (50%)	1 (10%)	
Cx	2 (10%)	1 (10%)	1 (10%)	
QCA post implantation				
Lesion length	18.51 ± 5.19^a^	16.77 ± 3.72	20.25 ± 6.01	0.137
Interpolated RVD	3.05 ± 0.54	2.82 ± 0.49	3.28 ± 0.50	0.056
MLD	2.71 ± 0.57	2.42 ± 0.60	2.99 ± 0.38	0.020
Diameter stenosis	11.70 ± 8.37	15.20 ± 9.50	8.20 ± 5.51	0.059
QCA at follow-up				
Lesion length	18.73 ± 5.26	17.43 ± 3.97	20.02 ± 6.25	0.284
Interpolated RVD	2.91 ± 0.52	2.66 ± 0.50	3.21 ± 0.37	0.020
MLD	2.59 ± 0.59	2.22 ± 0.60	2.95 ± 0.26	0.003
Diameter stenosis	13.39 ± 10.5	17.90 ± 11.65	7.75 ± 5.50	0.038
Late lumen loss	0.05 (0.02–0.31)^b^	0.11 (0.04–0.31)	0.03 (0.00–0.07)	0.165

*Cx* circumflex artery, *LAD* left anterior descending, *MLD* minimal lumen diameter, *RCA* right coronary artery

^a^Mean ± standard deviation. *t* test used for comparisons

^b^Median (interquartile range). Mann–Whitney test used for comparisons

### Quantitative OCT findings at 3-month follow-up

**Fig. 1:**
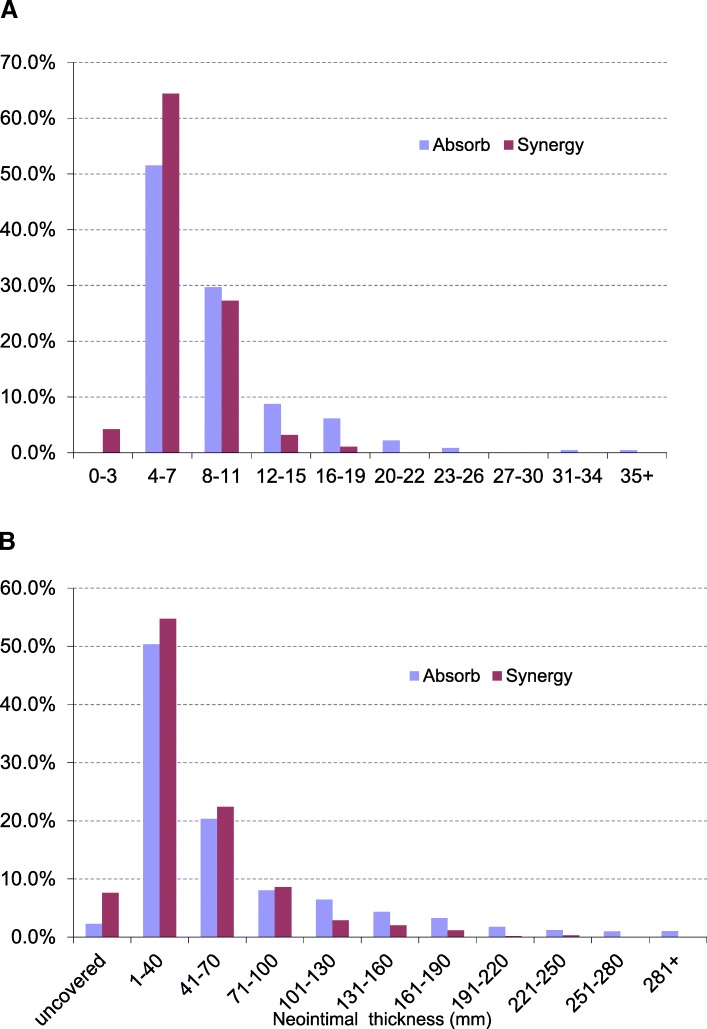
Histogram distribution of the in-stent/scaffold area obstruction and comparative neointimal thickness with DES vs BVS stents. **a** Histogram distribution of in-stent/scaffold area obstruction (%) per cross-sectional level (420 cross-sections). Everolimus drug-eluting stents (DES) had median (interquartile range) of 6.4% (5.2–8.4%) and everolimus bioresorbable vascular scaffolds (BVS) had median of 7.9% (6.6–10.5%), *p* <  0.001. **b** Histogram distribution of neointimal thickness at strut level (3942 struts). Everolimus DES had mean neointimal thickness (± SD) of 0.046 ± 0.038 mm and everolimus BVS had mean (± SD) of 0.061 ± 0.062 mm, *p* <  0.001

Table 4 presents the quantitative OCT findings at lesion-level analysis. The Synergy DES presented with a higher minimal lumen area (7.73 ± 2.12 mm^2^ vs 5.07 ± 2.00 mm^2^, *p* = 0.01) and a higher endoluminal stent area (8.13 ± 2.19 mm^2^ vs 5.39 ± 1.87 mm^2^, *p* = 0.008) than the everolimus BVS at 3-month follow-up. The Fig. 1 illustrates distribution of in-stent/scaffold area obstruction and neointimal thickness among the 2 groups.

**Table 4:** Stent-level analysis

	Absorb (*n* = 10)	Synergy (*n* = 10)	*p*
	*N* (%)	*N* (%)	
ROI length (mm)	23.28 ± 4.25^a^	21.03 ± 6.39	0.366
Proximal reference lumen area (mm^2^)	7.94 ± 2.89	11.15 ± 3.57	0.053
Distal reference lumen area (mm^2^)	7.85 ± 2.96	9.65 ± 3.22	0.209
Minimal lumen area (mm^2^)	5.07 ± 2.00	7.73 ± 2.12	0.010
Endoluminal neointimal	17.46 ± 6.91	14.42 ± 4.72	0.266
stent/scaffold volume (mm^3^)			
Abluminal neointimal + stent/scaffold strut volume (mm^3^)	44.34 ± 14.27	33.57 ± 10.94	0.074
Abluminal stent area (mm^2^)	7.03 ± 2.04	9.48 ± 2.47	0.027
Endoluminal stent area (mm^2^)	5.39 ± 1.87	8.13 ± 2.19	0.008
Malapposition volume > 0 mm^3^	2 (20%)	3 (30%)	1.000
Stent/scaffold with > 30% area stenosis	3 (30%)	0 (0%)	0.211
% area stenosis	18.4 + 15.8	7.6 ± 7.5	0.067
Neointimal thickness (mm)	0.04 (0.03–0.05)^b^	0.05 (0.04–0.06)	0.385
Maximal neointimal thickness (mm)	0.209 (0.173–0.245)	0.220 (0.140–0.260)	0.880
Maximal in-stent/scaffold obstruction (%)	11.5 (10–13)	11.5 (10–14)	0.970
In-stent/scaffold obstruction (%)	7.89 (6.84–9.42)	6.98 (6.26–7.33)	0.112
Neointima			
Homogeneous	6 (60%)	30 (30%)	
Heterogeneous	4 (40%)	70 (70%)	0.370
Disrupted stent/scaffold	1 (10%)	0	1.000

^a^Mean ± standard deviation

^b^Median (interquartile range)

*ROI* region of interest

Table 5 presents the OCT results at cross-sectional (420 cross-sections analysed) and strut (3942 struts analysed) levels. At the cross-sectional level, a higher total neointimal area (1.94 ± 0.57 mm^2^ vs 1.76 ± 0.60 mm^2^) as well as a higher in-stent/scaffold obstruction (9.6 ± 4.8% vs 7.0 ± 2.5%, *p* < 0.001) were observed in the Absorb arm. At the strut level, the neointimal thickness per strut (NIT) was lower in the Synergy group, even when 4-mm-diameter Synergy stents were excluded. Finally, at the patient level (Table 4), area stenosis tended to be lower with the everolimus DES (7.6 ± 7.5%) than with the everolimus BVS (18.4 ± 15.8%, *p* = 0.07).

**Table 5:** Cross-sectional and stent strut-level analysis

	All patients (*n* = 20)			Stent diameter < 4 mm^a^ (*n* = 15)		
	*N* (%)	*N* (%)	*p*	*N* (%)	*N* (%)	*p*
Cross-sectional analysis (number of struts)	Absorb (229)	Synergy (191)		Absorb (229)	Synergy (89)	
Lumen area (mm^2^)	6.61 ± 2.12^b^	9.48 ± 2.50	< 0.001	6.61 ± 2.12	7.67 ± 1.67	< 0.001
Adluminal stent/scaffold area (mm^2^)	8.52 ± 2.15	11.23 ± 2.72	< 0.001	8.52 ± 2.15	9.32 ± 1.74	0.002
Endoluminal stent/scaffold area (mm^2^)	6.65 ± 2.01	9.72 ± 2.48	< 0.001	6.65 ± 2.01	8.00 ± 1.60	< 0.001
Endoluminal neointimal area (mm^2^)	0.76 ± 0.29	0.76 ± 0.25	0.789	0.76 ± 0.29	0.73 ± 0.20	0.336
Abluminal neointimal area + stent/scaffold strut area (mm^2^)	1.94 ± 0.57	1.76 ± 0.60	0.002	1.94 ± 0.57	1.65 ± 0.35	< 0.001
Neointima						
Homogeneous	213 (93%)	162 (85%)		213 (93%)	79 (89%)	
Heterogeneous	16 (7%)	29 (15%)	0.007	16 (7%)	10 (11%)	0.214
Cross-section with intraluminal mass	3 (1.3%)	2 (1.1%)	1.000	3 (1.3%)	2 (2.3%)	0.622
Cross-section with malapposition area > 0	7 (3.1%)	6 (3.1%)	0.960	7 (3.1%)	0 (0.0%)	0.098
Cross-section with uncovered struts	43 (19%)	45 (24%)	0.230	43 (19%)	19 (21%)	0.603
Cross-section with malapposed and uncovered struts	0	1 (0.5%)	0.455	0	0 (0.0%)	
In stent/scaffold obstruction (%)	9.59 ± 4.81	7.01 ± 2.52	< 0.001	9.59 ± 4.81	8.12 ± 2.73	0.007
Stent strut level analysis	Absorb (2062)	Synergy (1880)		Absorb (2062)	Synergy (778)	
Uncovered struts (*n*, %)	47 (2.3%)	143 (7.6%)	< 0.001	47 (2.3%)	30 (3.9%)	0.021
Malapposed struts (*n*, %)	7 (0.3%)	23 (1.2%)	0.001	7 (0.3%)	1 (0.1%)	0.692
Malapposed and uncovered (*n*, %)	0 (0.0%)	4 (0.2%)	0.052	0 (0.0%)	0 (0.0%)	
Neointimal thickness per strut (mm^2^)	0.06 ± 0.06	0.05 ± 0.04	< 0.001	0.06 ± 0.06	0.05 ± 0.03	< 0.001

^a^Five patients with Synergy 4-mm-diameter stent excluded

^b^Mean ± standard deviation. *t test* used for comparisons

### Qualitative OCT findings at 3-month follow-up

**Fig. 2:**
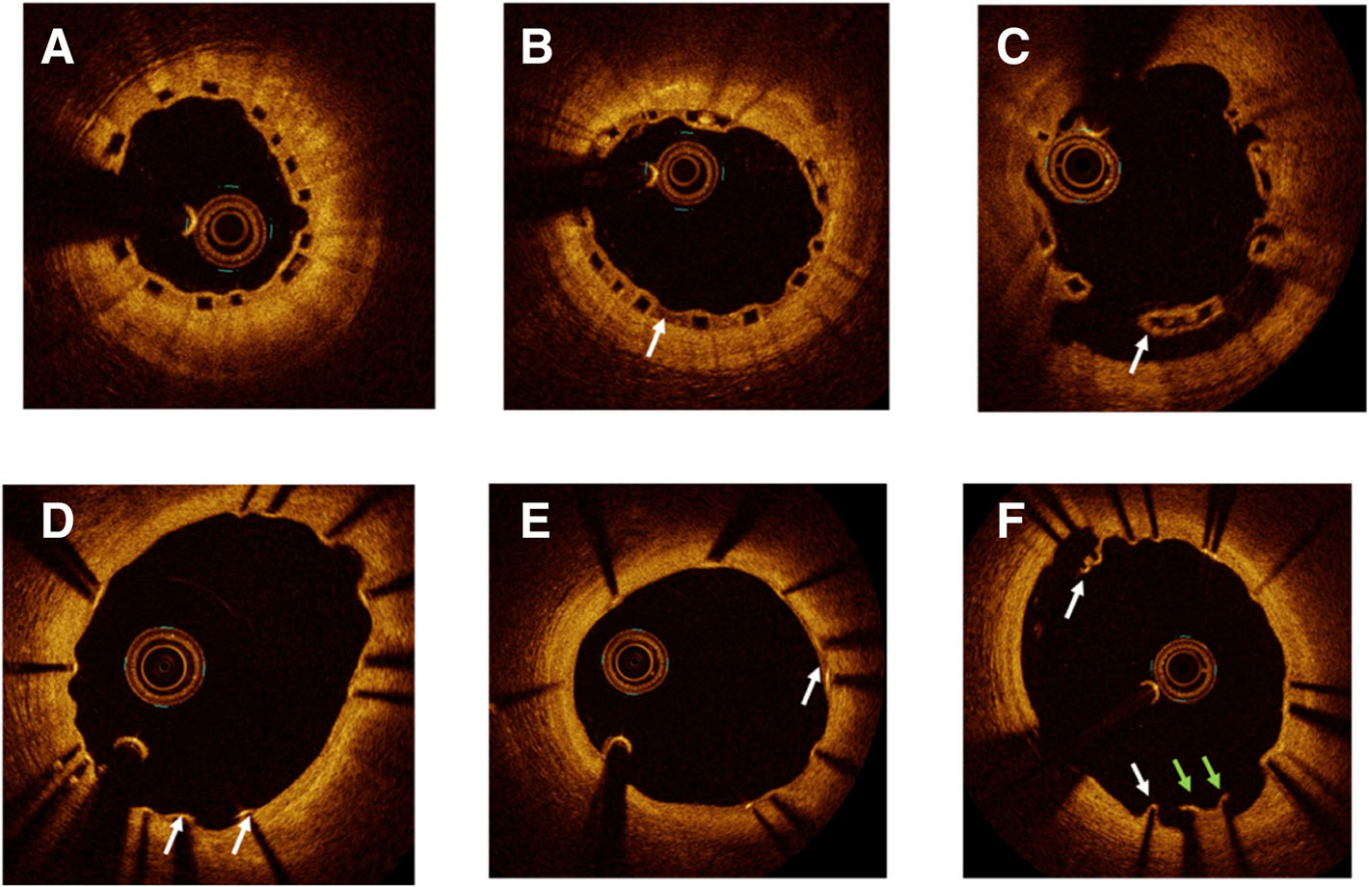
Qualitative OCT findings at 3-month follow-up. **a** Homogeneous neointima which covers Absorb (Abbott Vascular, Santa Clara, CA, USA) struts. **b** Heterogeneous neointima of Absorb (arrow). **c** Uncovered malapposed strut of Absorb (arrow). **d** Homogeneous neointima which covers the Synergy (Boston Scientific, Marlborough, MA, USA) struts, with two uncovered (arrows). **e** Heterogeneous neointima which covers Synergy struts (arrow). **f** Malapposed struts of covered (white arrows) and uncovered (green arrows) Synergy struts

Figure 2 and Table 5 present qualitative and quantitative OCT findings at 3-month follow-up. All struts had a preserved box appearance. At the strut level, the proportion of uncovered struts was lower in the Absorb arm (2.3% vs 7.6%, *p* < 0.001) and the difference was still significant when high-diameter Synergy stents were excluded. The proportion of malapposed struts was also higher in the Synergy group than in the Absorb group (1.2% vs 0.3% respectively, *p* < 0.001), but the difference did not remain when patients with high-diameter Synergy stents were excluded. There was a statistically significant trend to a higher rate of heterogeneous pattern of tissue coverage with the everolimus DES than with the everolimus BVS (15% vs 7%, *p* <  0.007).

### Clinical outcomes

Among the 20 patients who remained in the study until completion, device success was achieved in 19 patients (one patient in the BVS group had a residual stenosis of 32% post implantation). DOCE occurred in two patients (one patient in each group) subsequent to myocardial infarction. Two patients from the BVS group had a non-clinically driven TLR.

Between the 3-month and 12-month follow-up, no cardiac symptoms were observed. All 20 patients were alive at 1-year follow-up.

## Discussion

This trial is the first randomised clinical trial that compared the Absorb stent with the non-erodible metallic everolimus DES (Synergy with biodegradable polymer) while investigating stenting of culprit lesions in the setting of STEMI. Ideally, patients presenting with STEMI would represent the best scenario for using Absorb stents. Culprit lesions are frequently localised in the proximal segments of the coronary artery tree. Therefore, restoration of physiological vasomotion may have a greater effect in patients with STEMI, compared to patients with stable coronary artery disease. Finally, the potential advantages of implanting Absorb (vs other DES) in STEMI may be mostly related to the young age of these patients. Current evidence about the use of Absorb in STEMI remains scarce and limited to few registries that enlisted a low number of patients [15]. Recently, the results of the “ABSORB II” trial at 3-year follow-up, which only included non-MI patients [16], showed a higher rate of device-oriented composite endpoint in the Absorb group. Indeed, real-world randomised trials, observational registries, and meta-analysis suggest an approximate 3-fold incremental increase in scaffold thrombosis rates beyond 1 year after implantation of the Absorb BVS compared to the benchmark metal drug-eluting model [17]. On the other hand, the Synergy stent consists of a thin strut, balloon-expandable platinum–chromium stent platform delivering everolimus from an ultrathin (4 μm) bioabsorbable PLGA polymer applied to the abluminal surface [18]. Consequently, it was interesting to evaluate the very early re-endothelialisation of the Synergy stent vs the Absorb BVS in the setting of acute myocardial infarction. The principal findings can be summarised as follows: stent endothelisation on the basis of strut coverage was nearly complete for both devices, with more than 90% of covered struts at 3 months; after 3 months, the response of the arterial wall observed after stenting was different between the two groups, with lower neointimal thickness and in-stent obstruction in the Synergy group but with a lower rate of uncovered struts in the BVS group; the frequency of malapposed struts was lower in the Absorb arm; and at 3 months, late lumen loss tended to be lower in the Synergy arm.

In order to reduce the effect of stent diameter, OCT findings at 3 months were presented in the whole sample, but also after exclusion of patients treated with 4 mm-diameter Synergy stents. In both of these analyses, better results were obtained with Synergy stents in terms of lumen area and neointimal thickness of in-stent obstruction, but better results were obtained with BVS devices in terms of uncovered struts. Finally, our results suggest that the over-frequency of target-lesion failure or stent thrombosis observed with BVS in the Absorb III trial [19] may not be attributable to differences in endothelisation.

Our findings are significant for several other reasons. Rupture plaques in patients with STEMI have been shown to be prone to delayed arterial healing. Specifically, the mean rate of uncovered stents appeared to be as high as 49% in culprit lesions from STEMI, as compared to 9% in stable plaques after first-generation DES implantation [1]. The advent of second-generation DES has improved the arterial healing response. In an in vivo animal model, the use of everolimus eluting stent (EES) compared to first-generation sirolimus-eluting stent was associated with a lower incidence of uncovered struts and a minimal degree of inflammatory reaction [20]. These findings have been corroborated in humans in whom EES evidenced lower frequencies of uncovered struts and malapposed struts compared with paclitaxel-eluting stent [21], as assessed by OCT. Our results are promising, as they were obtained over a shorter follow-up period (3 months) and in the context of high thrombogenic milieu. Post implantation, MLD was higher in the Synergy group due to the absence of 4-mm-diameter stents in the Absorb group. Moreover, the higher post-dilatation approach in the Absorb trial (PSP technic) may explain the lower rate of uncovered and/or malapposed struts in this group. However, these two limitations do not modify the neointimal thickness and in-stent/scaffold area obstruction at 3-month follow-up. Finally, late loss was not significantly different between groups (0.10 vs 0.03 mm in the EES arm, *p* = 0.165).

## Study limitations

The study limitations include a monocentric trial and a low rate of events, which may be related to highly selective enrolment criteria (only non-complicated STEMI patients admitted during the recruitment period were included), inclusion and exclusion criteria, the requirement for patients’ consent in the acute phase of STEMI, the randomisation requirement after successful lesion preparation, and the requirement for an angiographic follow-up. Thus, the results presented herein cannot be representative of a more complex population suffering from STEMI.

Secondly, our study assessed arterial healing at 3 months, which is an intermediate time point when the resorption process is not complete and the process of neointima formation in EES has not yet occurred. A longer-term follow-up is needed to further characterise the healing process. Thirdly, healing-related results refer to the new Synergy stent technology and cannot be extrapolated to other bioresorbable devices with different materials or strut thickness. Finally, this randomised trial was an exploratory pilot study. In the absence of previous data on early re-endothelialisation after STEMI in the Synergy group, no hypothesis could be formulated on the differences to be expected in strut coverage between both groups at the time of the study design, and power computations were not possible.

## Conclusions

In this randomised trial conducted in the setting of MI, endothelisation was nearly complete with both the Synergy and the BVS devices. Some discrepancies were observed in the strut coverage rate (in favour of BVS) or in neointimal thickness (in favour of Synergy). This trial provides the basis for further exploration in clinical outcomes trials.

## Additional files

Additional file 1: SPIRIT 2013 checklist: recommended items to address in a clinical trial protocol and related documents (DOCX 61 kb)

Additional file 2: **Figure S1.** Schedule of enrolment, interventions, and assessments of the COVER-AMI study (DOCX 24 kb)
