# Supplementary material for: Re-endothelialisation after Synergy stent and Absorb bioresorbable vascular scaffold implantation in acute myocardial infarction: COVER-AMI study
Source: Trials. 2019 Apr 11;20:210. doi: 10.1186/s13063-019-3293-8 (PMC6458694; doi:10.1186/s13063-019-3293-8)
Supplement: Supplementary file 2 — Figure S1. Schedule of enrolment, interventions, and assessments of the COVER-AMI study (DOCX 24 kb) [file 13063_2019_3293_MOESM2_ESM.docx]

Figure S1: Schedule of enrolment, interventions, and assessments of the COVER-AMI study.

|  | **STUDY PERIOD** | | | | | | | |
| --- | --- | --- | --- | --- | --- | --- | --- | --- |
|  | **Enrolment** | **Allocation** | **Post-allocation** | | | | | **Close-out** |
| **TIMEPOINT** | ***-t_1_*** | **0** | ***t_1_*** |  | ***3-Month*** | ***1 year*** |  | ***1 year*** |
| **ENROLMENT:** |  |  |  |  |  |  |  |  |
| **Eligibility screen** | X |  |  |  |  |  |  |  |
| **Informed consent** | X |  |  |  |  |  |  |  |
| **Allocation** |  | X |  |  |  |  |  |  |
| **INTERVENTIONS:** |  |  |  |  |  |  |  |  |
| ***[BVS implantation]*** |  |  |  |  |  |  |  |  |
| ***[SYNERGY stent implantation]*** |  |  |  |  |  |  |  |  |
| **ASSESSMENTS:** |  |  |  |  |  |  |  |  |
| ***Baseline variables for inclusion and exclusion***  ***Inclusion criteria***  - Acute coronary syndrome with ST-elevation,  - One culprit lesion eligible for percutaneous coronary intervention (PCI) with stent implantation,  - Reference vessel measuring 2.5 mm to 3.75 mm per visual estimation, without extreme vessel tortuosity,  - TIMI 3 flow before stent deployment in the target vessel,  - Patient with at least 1 lesion eligible for planned PCI next to the culprit lesion,  - Patient aged between 18 and 85,  - Patient affiliated to the French national health care system (Sécurité Sociale),  - Patient agreed to participate after full information on the study (signature of an informed consent).  ***Exclusion criteria***  - Contra-indication to PCI and stent implantation,  - Contra-indication to prolonged dual antiplatelet therapy (defined by the combination of aspirin and a P2Y12 inhibitor),  - Known hypersensitivity to the active substance or to the excipients,  - Lesions located on coronary bypass or the left main coronary artery,  - Ostial lesions,  - Highly calcified lesions,  - Bifurcation lesions eligible to multiple stent implantation,  - History of stent Thrombosis,  - Cardiogenic shock,  - Renal failure(creatinine clearance < 30 ml/mn/1.73 m2),  - Breast-feeding,  - Pregnancy,  - Adult protected by the law (« tutelle », « curatelle » or « sauvegarde de justice » as designed by the French law),  - Patient participating in another biomedical research.  ***Baseline variables***  Age (years)  Male  Treatment for hypertension  Treatment for hypercholesterolemia  Diabetes mellitus  Current smoking  MDRD clearance < 60 ml/mn/1.73m^2^  Fasting plasma glucose ≥ 7 mmol/l  LVEF (%)  LAD Significant lesion (> 50 %)  Cx Significant lesion (> 50 %)  RCA Significant lesion (> 50 %)  > 1 vessel disease  **Vessel**  RCA  LAD  Cx  **QCA post implantation**  Lesion length  Interpolated RVD  MLD  Diameter stenosis  **QCA at follow-up**  Lesion length  Interpolated RVD  MLD  Diameter stenosis  Late Lumen Loss | X | X |  |  |  |  |  |  |
| ***Angiographic and OCT outcome variables***  ***Angiographic data***  Lesion length  Interpolated RVD  MLD  Diameter stenosis  Late Lumen Loss  **OCT data**  Uncovered struts (n, %)  Malapposed struts (n, %)  Malapposed and uncovered (n, %)  Neointimal thickness per strut (mm^2^) |  |  |  |  | X |  |  |  |
| ***Procedural and clinical events***  Device success, death, myocardial infarction, target vessel revascularization, target lesion revascularization, stent thrombosis |  |  | X |  | X | X |  | X |
